# Deletion lengthening at chromosomes 6q and 16q targets multiple tumor suppressor genes and is associated with an increasingly poor prognosis in prostate cancer

**DOI:** 10.18632/oncotarget.22408

**Published:** 2017-11-11

**Authors:** Martina Kluth, Simon Jung, Omar Habib, Mina Eshagzaiy, Anna Heinl, Nina Amschler, Sawinee Masser, Malte Mader, Frederic Runte, Philipp Barow, Sohall Frogh, Jazan Omari, Christina Möller-Koop, Claudia Hube-Magg, Joachim Weischenfeldt, Jan Korbel, Stefan Steurer, Till Krech, Hartwig Huland, Markus Graefen, Sarah Minner, Guido Sauter, Thorsten Schlomm, Ronald Simon

**Affiliations:** ^1^ Institute of Pathology, University Medical Center Hamburg-Eppendorf, Hamburg, Germany; ^2^ Genome Biology Unit, European Molecular Biology Laboratory (EMBL), Heidelberg, Germany; ^3^ Martini-Clinic, Prostate Cancer Center, University Medical Center Hamburg-Eppendorf, Hamburg, Germany; ^4^ Department of Urology, Section for Translational Prostate Cancer Research, University Medical Center Hamburg-Eppendorf, Hamburg, Germany

**Keywords:** prostate cancer, deletion lengthening, 6q, 16q, tissue microarray

## Abstract

Prostate cancer is characterized by recurrent deletions that can considerably vary in size. We hypothesized that large deletions develop from small deletions and that this “deletion lengthening” might have a “per se” carcinogenic role through a combinatorial effect of multiple down regulated genes. *In vitro* knockdown of 37 genes located inside the 6q12-q22 deletion region identified 4 genes with additive tumor suppressive effects, further supporting a role of the deletion size for cancer aggressiveness. Employing fluorescence *in-situ* hybridization analysis on prostate cancer tissue microarrays, we determined the deletion size at 6q and 16q in more than 3,000 tumors. 16q and 6q deletion length was strongly linked to poor clinical outcome and this effect was even stronger if the length of both deletions was combined. To study deletion lengthening in cancer progression we eventually analyzed the entire cancers from 317 patients for 6q and 16q deletion length heterogeneity and found that the deletion expanded within 50-60% of 6q and 16q deleted cancers. Taken together, these data suggest continuous “deletion lengthening” as a key mechanism for prostate cancer progression leading to parallel down regulation of genes with tumor suppressive properties, some of which act cooperatively.

## INTRODUCTION

Chromosomal deletions are a common feature of human solid cancers. Almost 50 years ago, Knudson demonstrated that deletions often represent one of the two “hits” required for biallelic inactivation of specific tumor suppressor genes residing inside the deleted region [1, 2]. Current genomics studies and functional screens have now revealed another role of deletions, which may be particularly effective in large chromosomal defects: Most large deletions are heterozygous and lack a recurrent gene-specific second hit, but highlight genomic loci enriched for so called “STOP” genes with tumor suppressive properties [3, 4]. This led to the hypothesis that compound [5] or cumulative [4] haplo-insufficiency, i.e. concurrent down-regulation of multiple genes by large heterozygous deletions, is an important driving force in deletion-rich cancers.

Prostate cancer is a prime example for a deletion-rich cancer lacking recurrent second hits. Using whole genome copy number analysis [6-8] and whole genome sequencing [9-11], others and us have identified recurrent deletion regions affecting up to 40% of these tumors, while somatic non-silent mutations typically occur only in less than 5% of prostate cancers. Virtually all deletion regions are characterized by a marked variability of the size of deleted chromosomal segment [6-8] and some studies have demonstrated a strong link between a cancer`s deletion burden and adverse patient outcome [6, 12]. We hypothesize that the variable size of most deletions reflects a continuous process involving progressive loss of chromosomal material, which parallels increasing tumor aggressiveness.

To test this hypothesis, we made use of our unique resource of more than 7,000 annotated clinical prostate cancer samples and applied fluorescence in-situ hybridization (FISH) and functional analysis to detect growing deletions and consequences of multiple tumor suppressor gene inactivation at two hot spot deletion areas at chromosomes 6q and 16q.

## RESULTS

### Multiple tumor suppressor genes (TSG) are co-targeted by large deletions

**Figure 1:**
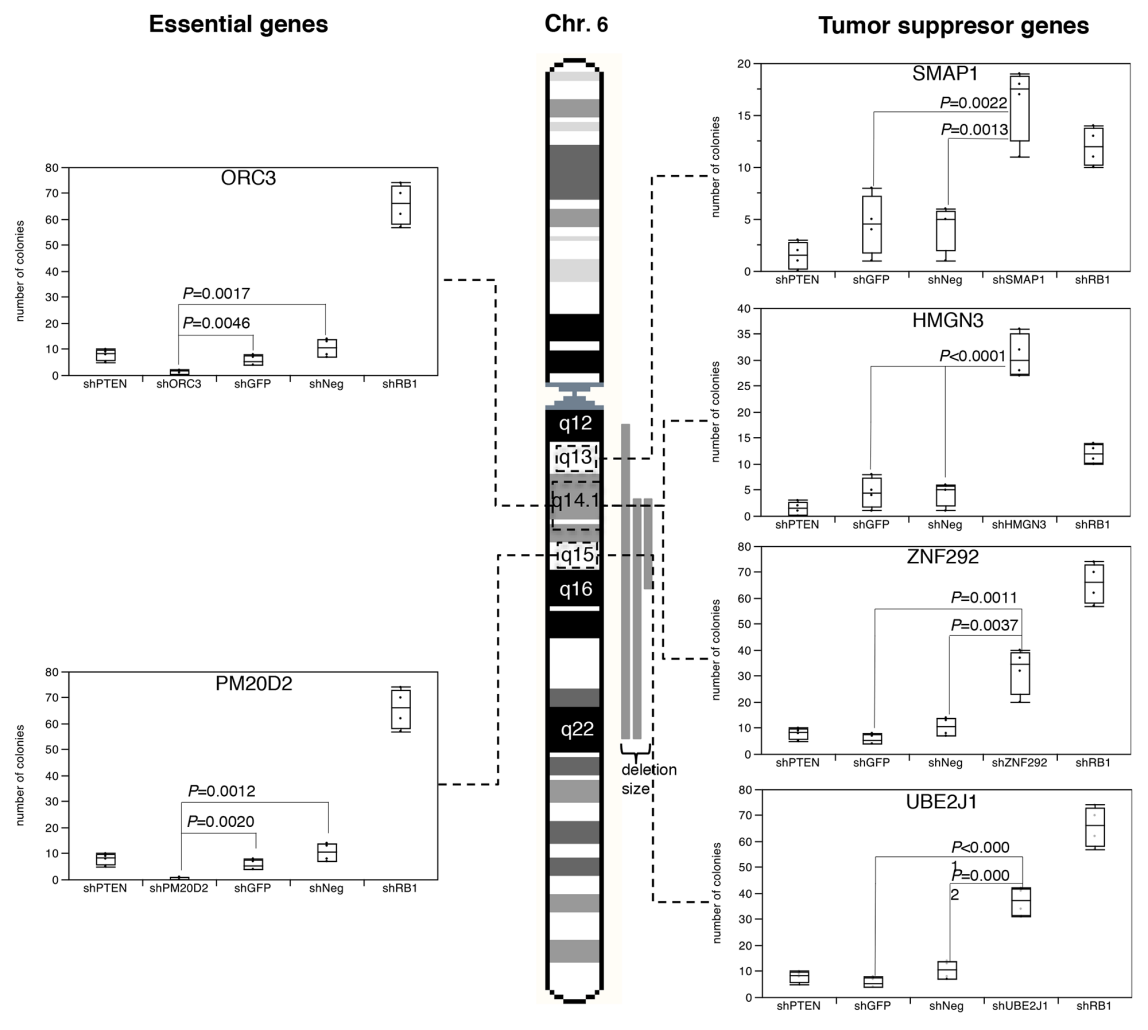
Results of the colony formation assay after shRNA-based depletion of UBE2J1, ZNF292, SMAP1, HMGN3, PM20D2 and ORC3 in BPH-1 cells Controls included shPTEN and shRB1 as positive control, as well as shNeg and shGFP for negative control.

In order to test whether multiple tumor relevant genes reside in the large 6q12-q22 deletion, we selected 37 genes that were expressed in DU145, PC-3 and BPH-1 and had a known function that was compatible with a TSG, and subjected them to shRNA-mediated depletion in PC-3, DU145, and BPH-1 cells (Supplementary Figure 1). We found that down regulation of each of 4 genes, i.e., *SMAP1* (6q13), *ZNF292* (6q14), *HMGN3* (6q14), and *UBE2J1* (6q15) significantly increased cell growth over background in colony formation assays performed in duplicate (*P* ≤ 0.0139, Figure 1 and Supplementary Figure 2), thus supporting a tumor suppressive role for these four genes. *UBE2J1* encodes an E2 ubiquitin-conjugating enzyme that contributes to degradation of proteins targeted by E3 ubiquitin ligases [13]. The zinc-finger protein ZNF292 is a putative regulator of transcription according to the Gene Ontology (GO) database. Inactivation of SMAP1, a GTPase-activating protein specific for ADP-ribosylation factor 6 acting on membrane trafficking and actin remodeling has been suggested to contribute to development of microsatellite instability associated oncogenesis [14]. HMGN3 is a member of the high motility group of chromatin-binding proteins involved in DNA unwinding, repair, and transcription control [15].

In contrast, depletion of 2 genes (*ORC3*, *PM20D2*) virtually completely abolished cell growth (Figure 1). *ORC3* encodes for subunit of the origin recognition complex, which is essential for the initiation of the DNA replication in eucaryotic cells [16]. PM20D2 is, by homology, a putative hydrolase, which may play a role in metabolic processes. Heterozygous deletion of essential genes has been postulated to render cancer cells vulnerable to further inhibition of these genes [17], and more than 50 genes have been identified suppression of which specifically inhibited the proliferation of cells harboring partial copy number loss of these genes [18]. Interestingly, such essential genes had been suggested as promising targets for anti-cancer therapies, and were thus termed CYCLOPS (copy number alterations yielding cancer liabilities owing to partial loss) genes [18]. Our data suggest that also *ORC3* and *PM20D2* may represent CYCLOPS genes that could potentially serve as future drug targets in prostate cancers harboring large 6q deletions.

### At least four genes drive growth of cancer cells harboring large 6q deletions additively

**Figure 2:**
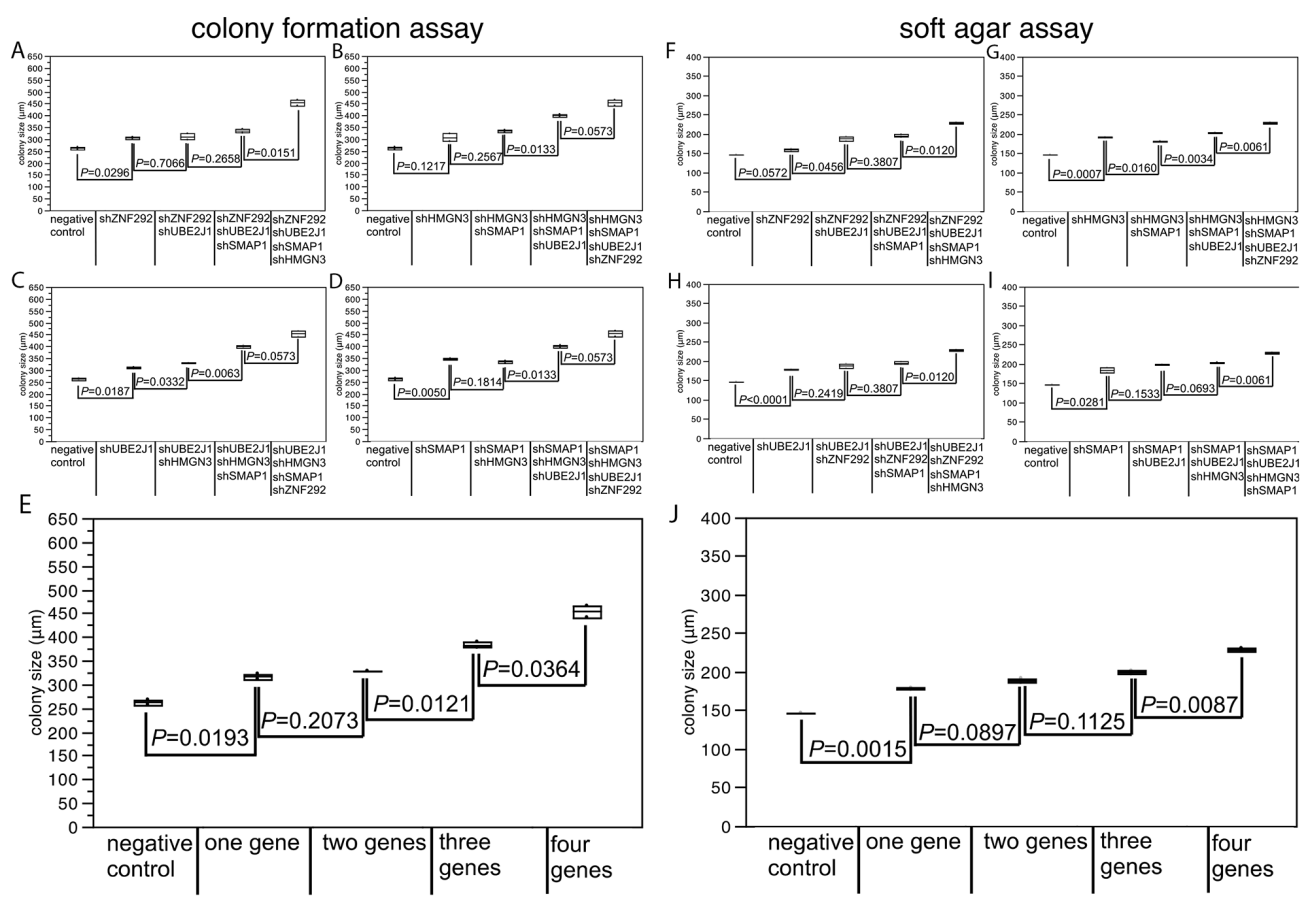
Effect of co-depletion of candidate tumor suppressor genes in DU145 cells on **(A-E)** colony size in the colony formation assay, **(F-J)** colony size in the soft agar assay. Average colony size of all analysis are mapped in figure (E) for colony formation assay and in figure (J) for soft agar assay.

To assess whether the 4 newly identified putative 6q TSGs cooperatively affect cell growth, anchorage independent growth, or motility, we performed co-depletion experiments of these genes in DU145 cells (Figure 2). In order to mimic the haplo-insufficient state resulting from heterozygous deletion to the best possible extent, we selected experimental conditions that resulted in a knock-down efficacy of 50-70% relative to the control (Supplementary Figure 3B). In a colony formation assay, the colony size increased with the number of silenced genes (Figure 2E), although not all differences were statistical significant. That this effect was independent from specific combinations of co-depleted genes (Figure 2A-2D) demonstrates that each of the four genes contributed equally to the accumulative loss of growth control.

Confirmatory results were obtained from the soft agar assay, where co-depletion of all four candidate TSGs resulted in the strongest increase in colony size (*P* ≤ 0.0146; Figure 2J). No impact of cell motility was found for any of the tested genes (Supplementary Table 1). In line with our findings, a recent study has shown that inactivating *SMAP1* mutations increased cell clonogenicity and cell proliferation by shortening the G2/M phase [14]. HMGN3 is a regulator of a plethora of genes, some of which are involved in glucose and fat metabolism, including the putative tumor suppressor gene *AZGP1*, inactivation of which has been linked to tumor progression, poor prognosis, and increased cell proliferation in prostate cancer before [19]. Taken together, these data suggest that simultaneous reduction of the activity of pivotal genes results in a critical loss of growth control in tumors carrying large 6q deletions. Of note, finding 4 tumor relevant genes among the 37 genes analyzed in our functional screen suggests that several additional genes with tumor suppressive features may be present among the remaining 140 genes that reside in the large deletion region and may induce additional synergistic effects on cells.

### Increased deletion size is associated with aggressive tumor features

**Figure 3:**
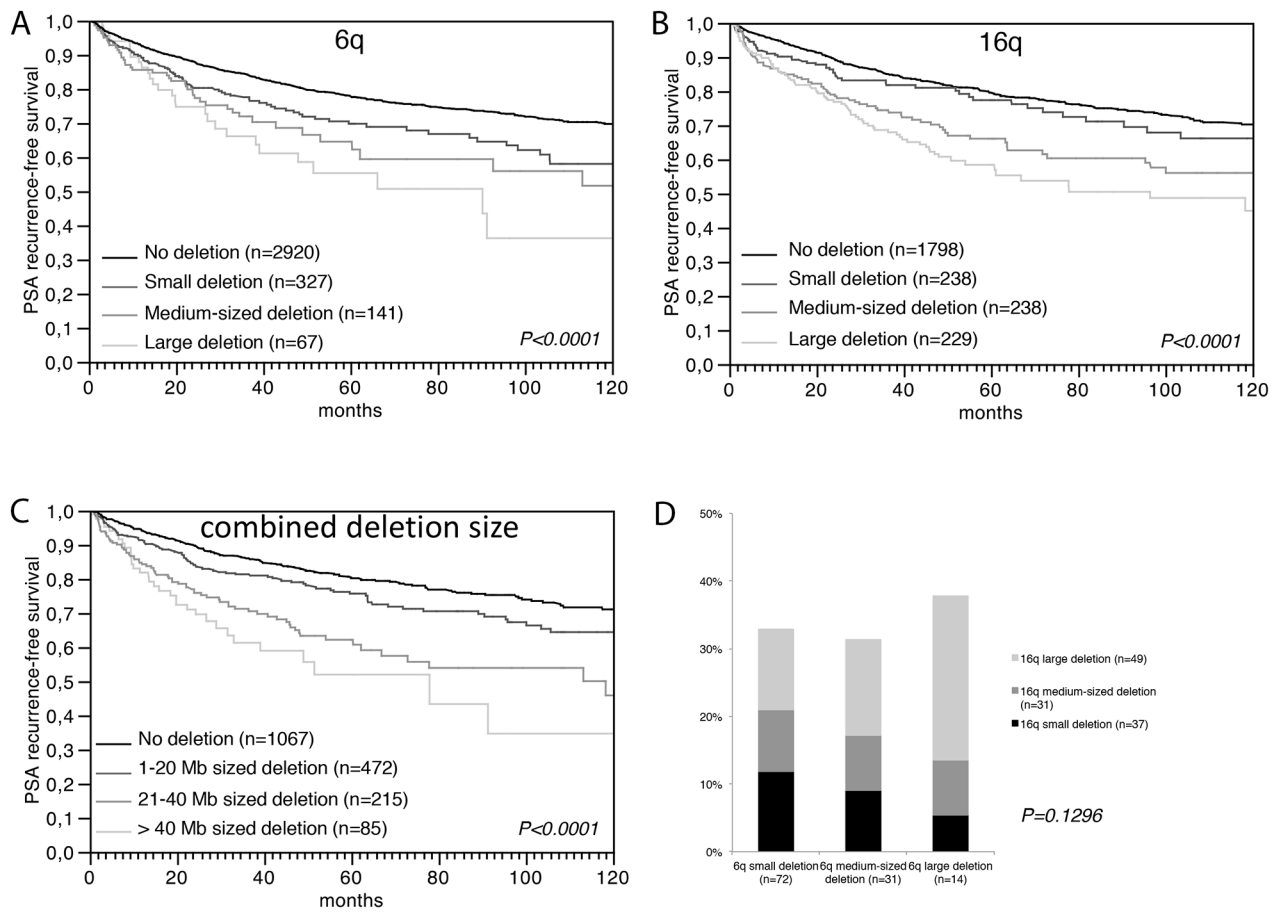
Prognostic relevance of **(A)** the 6q deletion size, **(B)** the 16q deletion size, and **(C)** the combined 6q and 16q deletion size. **(D)** Association between the size of 6q and 16q deletions. 6q deletion size: small=6q14-16 or 6q15, 10 mega bases (Mb); medium=6q14-22, 40 Mb; large=6q12-22, 55 Mb. 16q deletion size: small=6q24, 3 Mb; medium=6q23-24, 12 Mb, large=6q21-24, 30 Mb.

We next tested the hypothesis, that large deletions have more clinical impact than a more limited loss of chromosomal material. We analyzed a prostate cancer tissue microarray (TMA) containing one 0.6 mm tissue spot each from 7,433 different patients with 8 sets of FISH probes, including 5 probes located at 6q (6q12, 6q14, 6q15, 6q16, and 6q22) as well as 3 probes at 16q (16q21, 16q23, and 16q24), in order to determine the size of deletions at these loci. If all probes were analyzed separately, we found that all losses were heterozygous, most frequent at the typical regions of the minimal common deletion at 6q15 (19%) and 16q24 (28%) [6, 20-23], and that the deletion frequency declined with growing distance from these hotspots (i.e. 17% at 6q14 or 6q16, 8% at 6q22, and 3% at 6q12, as well as 21% at 16q23 and 10% at 16q21, Tables 1, 2). These figures fit well to the published frequency and size distribution of these deletions (Supplementary Figure 4), thus demonstrating the validity of our FISH analysis and scoring. Comparing our deletion data with the pathological and clinical information attached to our TMA, we found that the presence of deletions was linked to adverse tumor features largely irrespective of the individual chromosomal locus that was analyzed. This was equally true for associations between deletions at 6q12, 6q14, 6q15, 6q16 or 6q22 and advanced tumor stage and high Gleason grade (all data and *P*-values summarized in Table 1) as well as for deletions at 16q21, 16q23, or 16q24 and advanced tumor stage, high Gleason grade, presence of lymph node metastases, and presence of a positive surgical margin (summarized in Table 2). To investigate whether the deletion size had clinical impact beyond the sole presence of deletions, we considered only the subsets of tumors with interpretable results for all FISH probes at 6q (n=3,725) and 16q (n=2,712), and categorized the tumors according to the deletion size. Among tumors with 6q deletions (573/3,725), there were 74 (12.9%) tumors with large (6q12-q22), 151 (26.4%) tumors with medium (6q14-q22) and 348 (60.7%) tumors with small deletions (6q14-q16). Amoung tumors with chromosome 16q deletion (763/2,712), we identified 251 (32.9%) tumors with large (16q21-q24), 254 (33.3%) with medium (16q23-q24), and 258 (33.8%) with small deletions (16q24). At 16q, increasing deletion size strongly paralleled an increase of the tumor stage (*P* < 0.0001) and Gleason grade (*P* = 0.0019, Table 2). No such associations were found for deletions at 6q (Table 1). However, the deletion size at both loci had a strong impact on patient prognosis. Tumors showing large deletions at 6q or at 16q had the shortest relapse-free interval, while tumors with small deletions limited to 6q14-q16 or 16q24 had the longest. An intermediate outcome was found for patients with medium-sized deletions at 6q14-q22 or 16q23-q24 (each *P* < 0.0001) (Figure 3A, 3B). The comparatively good prognosis of cancers with small 6q15 deletions observed in our study is also in line with our observation in the initial functional screen that an unequivocal tumor suppressive role could not be seen for any of the five genes (*MDN1*, *CASP8AP2*, *GJA10*, *BACH2*, *MAP3K7*) that had earlier been located to the deletion epicenter at 6q15 in prostate cancer before [6, 23-26]. A comparable observation to our data has recently been made in chronic lymphatic leukemia, a hematologic cancer that is frequently characterized by focal heterozygous deletion of the *DLEU2* (deleted in lymphocytic leukemia 2) gene locus at 13q14. Dal Bo et al. reported that tumors with larger deletions involving also the *RB1* gene located 1.8 megabases centromeric from *DLEU2* had a worse prognosis than those with small *DLEU2* deletions [27]. Together with our data, these findings strongly support the hypothesis that large deletions are a key mechanism for compound haplo-insufficiency [5] with loss of tumor suppressive capabilities in prostate cancer.

**Table 1:** Associations of 6q deletions at different loci and of the deletion size with clinico-pathological parameters of prostate cancer

		All cancers	Tumor stage			Gleason grade				Lymph node metastasis		Surgical margin	
			pT2	pT3a	pT3b	≤3+3	3+4	4+3	≥4+4	N0	N+	Negative	Positive
**6q12**	**analyzable (n)**	3493	2182	831	464	975	1818	520	157	1921	170	2724	715
	**deletion (%)**	3.1	2.9	2.8	4.5	1.8	3.0	5.0	4.5	3.2	4.7	3.0	3.5
	**P-value**		0.1822			0.0070				0.3297		0.4782	
**6q14**	**analyzable (n)**	3514	2177	856	467	989	1812	535	158	1946	178	2736	722
	**deletion (%)**	17.7	16.3	18.3	22.9	10.5	17.3	28.8	27.2	19.7	18.5	17.4	18.1
	**P-value**		0.0031			<0.0001				0.7115		0.6568	
**6q15**	**analyzable (n)**	3987	2514	948	506	1151	2052	586	172	2238	181	3103	818
	**deletion (%)**	18.8	17.1	20.4	24.5	10.9	18.7	31.2	30.2	20.7	24.9	18.6	18.9
	**P-value**		0.0003			<0.0001				0.1933		0.8012	
**6q16**	**analyzable (n)**	3514	2177	856	467	989	1812	535	158	1946	178	2736	722
	**deletion (%)**	17.2	15.7	18.1	22.1	10.3	16.7	28.0	25.9	19.0	18.0	17.1	16.5
	**P-value**		0.0037			<0.0001				0.7466		0.6907	
**6q22**	**analyzable (n)**	3493	2182	831	464	975	1818	520	157	1921	170	2724	715
	**deletion (%)**	8.5	7.2	10.0	11.9	5.1	8.6	12.9	12.7	9.3	11.8	8.1	9.4
	**P-value**		0.0013			<0.0001				0.3008		0.3009	
**Deletion size**	**analyzable (n)**	573	321	151	98	90	291	143	42	347	35	442	120
	**small (6q14-q16) (%)**	60.7	63.6	58.9	56.1	61.1	61.5	60.8	59.5	62.0	60.0	61.5	60.0
	**medium (6q14-q22) (%)**	26.4	24.0	30.5	27.6	25.6	27.1	25.2	26.2	25.9	28.6	26.2	25.8
	**large (6q12-q22) (%)**	12.9	12.5	10.6	16.3	13.3	11.3	14.0	14.3	12.1	11.4	12.2	14.2
	**P-value**		0.4041			0.9887				0.9445		0.8526	

**Table 2:** Associations of 16q deletions at different loci and of the deletion size with clinico-pathological parameters of prostate cancer

		All cancers	Tumor stage			Gleason grade				Lymph node metastasis		Surgical margin	
			pT2	pT3a	≥pT3b	≤3+3	3+4	4+3	≥4+4	N0	N+	Negative	Positive
**16q21**	**analyzable (n)**	2848	1728	737	372	730	1526	462	114	1604	144	2194	598
	**deletion (%)**	10.1	8.0	12.9	13.4	4.9	9.0	18.2	21.1	9.9	18.8	9.5	11.7
	**P-value**		<0.0001			<0.0001				<0.0001		0.1134	
**16q23**	**analyzable (n)**	3831	2366	956	489	1051	2008	597	148	2092	188	2960	806
	**deletion (%)**	21.2	17.3	24.1	33.9	13.4	20.4	33.2	35.1	21.6	36.7	19.8	25.6
	**P-value**		<0.0001			<0.0001				<0.0001		0.0004	
**16q24**	**analyzable (n)**	2846	1727	737	371	730	1526	460	114	1603	144	2194	596
	**deletion (%)**	27.9	23.5	32.8	37.7	17.9	27.5	40.7	41.2	28.1	40.3	26.5	31.5
	**P-value**		<0.0001			<0.0001				0.0270		0.0152	
**Deletion size**	**analyzable (n)**	763	391	234	132	124	404	181	45	433	57	557	181
	**small (16q24) (%)**	33.8	40.4	28.2	25.8	49.2	35.9	23.8	20.0	33.3	19.3	35.4	30.4
	**medium (16q23-q24) (%)**	33.3	28.6	35.9	42.4	28.2	33.4	36.5	33.3	34.6	38.6	32.1	36.5
	**large (16q21-q24) (%)**	32.9	30.9	35.9	31.8	22.6	30.7	39.8	46.7	32.1	42.1	32.5	33.1
	**P-value**		<0.0001			0.0019				0.0752		0.4083	

### Large co-deletions are linked to a particularly poor patient prognosis

We next tested whether the amount of loss of genomic material from multiple different chromosomes and associated “cross-chromosomal compound haplo-insufficiency” might cause increased malignancy (Figure 3D). Employing an “*in silico*” approach, our clinical follow-up data were compared to the accumulated deletion size at both chromosomes. For this purpose, we estimated the deletion size in each individual tumor at 6q and at 16q based on the distance in mega base (Mb) pairs between the FISH probes indicating deletion, and summarized the total deletion length at 6q and 16q in each tumor. Comparison with clinical data revealed a strong relationship between the cumulative deletion size and shortened prostate specific antigen (PSA) recurrence-free interval. Tumors with a combined 6q and 16q deletion size of more than 40 Mb had the worst prognosis, while tumors with a deletion size between 1-20 Mb had the best prognosis (*P* < 0.0001, Figure 3C). A multivariate analysis demonstrated that the prognostic value of the deletion size was independent from the established prognostic markers Gleason grade, tumor stage, nodal stage, resection margin status, and preoperative PSA level (Supplementary Table 2). These findings support a model in which cancer aggressiveness is connected to the total amount of deleted chromosomal material and, therefore, to the number of genes affected by these deletions. Similar results have been obtained by Hieronymus et al. who demonstrated that the overall number of copy number alterations in a prostate cancer genome is linked to patient outcome [12]. Given that the likelihood for synergistic hits increases with the number of deleted genes, we sought for possible interactions between genes at 6q and 16q, and performed a gene enrichment analysis including our 6q candidate TSG as well as 16q candidate genes obtained from the literature, such as *MAF* [28], *ATBF1* [29], *FOXF1* [28], *MVD* [28], *WFDC1* [28, 30], *WWOX* [28, 31], *CDH1* [32], and *CRISPLD2* [33]. We found 4 genes (*CRISPLD2* at 16q24.1, *CDH13* at 16q23.3, and *MAF* at 16q22, as well as *HMGN3* at 6q14.1), which are part of a set of genes up regulated in the urogenital sinus (the embryonal origin for the developing prostate) in mice exposed to the androgen dihydrotestosterone [34]. During embryogenesis, prostate epithelial cells develop from the urogenital sinus by proliferation and invasion of the surrounding urogenital sinus mesenchyme. It is, thus, tempting to speculate that one possible functional consequence of large co-deletions of 6q and 16q is targeting of androgen-responsive cellular pathways connected to maintenance of normal prostate cell differentiation.

### Large deletions develop through progressive loss of chromosomal material

To investigate whether tumors with large and small deletions arise de-novo, or if large deletions develop from progressive loss of adjacent chromosomal material in tumors initially carrying small deletions, we expanded our 6q and 16q FISH analyses to a heterogeneity TMA containing 10 samples taken as distant as possible from each other from each of 317 prostate cancers. 6q and 16q deletion analysis led to informative data in 208 and 182 patients in which at least three cancer containing tissue spots were analyzable for all FISH probes, including 5 probes located at 6q (6q12, 6q14, 6q15, 6q16, and 6q22) as well as 3 probes at 16q (16q21, 16q23, and 16q24). For deletion development analysis, we selected 51 cancers each that had deletions at 6q or 16q in at least three cancer spots.

Deletions of constant size across all deleted cancer spots were found in 21 (41.2%) of 51 cases for 6q and 22 (43.1%) of 51 cases for 16q. That 11 (6q) and 15 (16q) of these tumors had exclusively large deletions (i.e., 6q14-q22 or larger, 16q23-q24 or larger) suggests that large deletions either can develop de novo or that potential tumor areas with smaller progenitor deletions have been missed.

**Figure 4:**
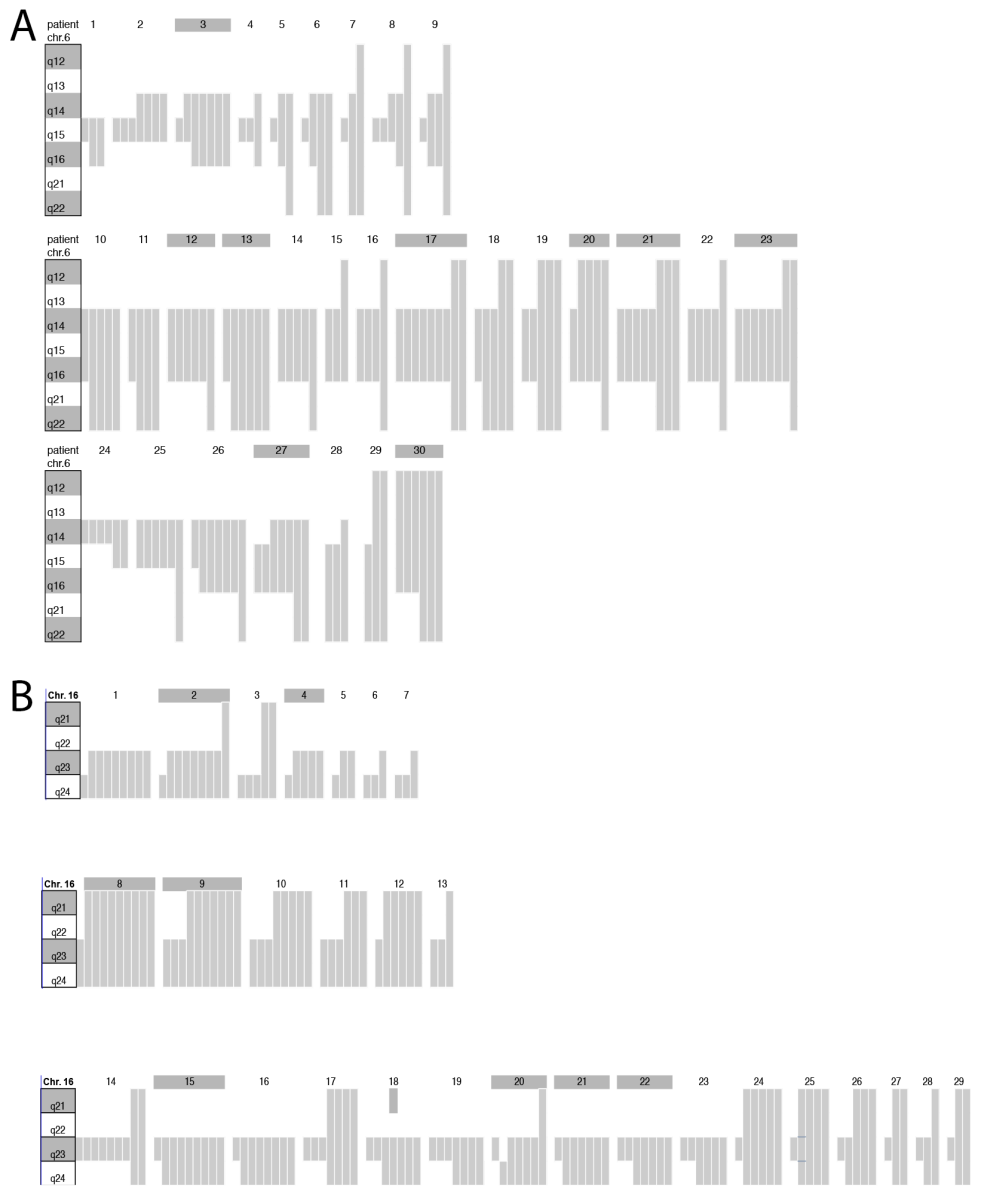
Schematic plot of the 6q and 16q deletion size heterogeneity determined by FISH analysis in the tumors of 30 and 29 patients **(A)** Chromosome ideograms display the 6q12-q22 region. Gray bars represent the size of the 6q deletion found in each of up to 10 different tissue spots that were analyzed per tumor. Spots without deletion are not shown. Shaded patient numbers indicate cases with 6q deletion in all analyzable tissue spots. **(B)** Chromosome ideograms display the 16q21-q24 region. Gray bars represent the size of the 16q deletion found in each of up to 10 different tissue spots that were analyzed per tumor. Spots without deletion are not shown. Shaded patient numbers indicate cases with 16q deletion in all analyzable tissue spots.

Deletions of variable size were found in 30 (58.8%) and 29 (56.9%) cases for 6q and 16q (Figure 4). Remarkable, the 6q deletion patterns revealed that virtually all (29 of 30) deletions originated from 6q15. In contrast, analysis of 16q identified two regions of origin, i.e., 16q23 in 17 cases and 16q24 in 7 cases. Taken together, our data demonstrates that about 50-60% of 6q and 16q deletions develop as small losses and then increase in size by subsequent loss of adjacent chromosomal material. The molecular mechanisms driving 6q and 16q deletion lengthening remain to be elucidated. It could be speculated that the same mechanisms driving breakage and fusion of the *TMPRSS2:ERG* loci, i.e., chromatin movements induced by androgen receptor (AR) signaling that predispose specific chromosomal loci to double strand breakage and translocation [35], could also account for breakage and interstitial deletion. Changes in the chromosome structure induced by small deletion might include rapprochement of normally remote AR binding sites that could potentially predispose for additional AR-driven breakage with loss of genetic material.

## DISCUSSION

In this study, we demonstrate that the size of heterozygous deletions in human prostate cancer greatly correlates with tumor progression and aggressiveness. That we made this observation in two of the most frequently deleted loci, i.e., 6q and 16q, and that the vast majority of deletions in prostate cancer typically affect large chromosomal segments [6-8, 22], suggests that extensive loss of chromosomal material by progressive “deletion lengthening” is a key mechanism for simultaneous dosage reduction of multiple genes. Co-depletion of arbitrarily selected candidate tumor suppressor genes located inside the large 6q12-q22 deletion region identified 4 genes, including *UBE2J1*, *ZNF292*, *SMAP1*, and *HMGN3* as a prime example for assumedly many more genes that jointly drive tumor growth when their activity is reduced by large heterozygous deletions. In line with our findings, cooperative effects have also been reported from mouse orthologs of human 8p11-p23 genes in a mouse model of HCC [36]. Of note, large 8p deletion, often involving the entire chromosome arm, is one of the most frequent alterations in many solid tumor types including prostate cancer [6-8, 37]. Moreover, using an “in silico” approach to integrate clinical and molecular data obtained from our large tumor set before, we demonstrate that such cooperative effects are not limited to genes located inside the same deletion, but are also effective in co-deletions located at different chromosomes. Mechanistically, our observations support a model of increasing likelihood for cooperative tumor promoting effects with increasing deletion size, but strongly argue against the concept that one particular tumor suppressor gene drives the development of large deletions. Thus, our findings explain why the “classical” approach of tumor suppressor gene identification in chromosomal regions of deletion, i.e. searching for a gene with a “2^nd^ hit” inside the minimal commonly deletion region, has not been successful in prostate cancer [10].

Our study also highlights novel starting points for diagnostic and therapeutic approaches specifically connected to the presence of large deletions. We found that both the deletion size as well as the number of deletions per tumor had a strong predictive value independently from the classical prognostic factors including tumor stage, Gleason grade, and nodal stage. While determination of the accumulated deletion lengths per cancer requires whole genome analysis assays that are expensive and time consuming, FISH analysis for multiple defined loci can be comparatively easy performed on routine diagnostic tissue samples including also preoperative punch biopsies. A therapeutic approach might arise from the detection of essential genes, e.g., *ORC3* and *PM20D2*, in the large 6q deletion region. Obviously, presence of essential genes provides an explanation for the exclusively heterozygous nature of large deletions, since these genes must not be completely inactivated (for example, by homozygous deletion) in order to allow tumor cells to survive. Given that such essential genes inside region of heterozygous deletion have been postulated to render cancer cells vulnerable to further inhibition of these genes [17], the association of large deletions with advanced and aggressive cancers observed in our study justifies further research on deletion-based therapeutic strategies against prostate cancer in men.

## MATERIALS AND METHODS

### Patients and tissue microarrays

Two TMAs, including a prostate cancer prognosis TMA, and a prostate cancer heterogeneity TMA were used in this study. The prostate cancer prognosis TMA was expanded from a previous version containing one tumor sample of 3,261 radical prostatectomy specimens [38] by adding additional 4,634 cancers. Clinical follow-up data were available for 6,870 of the 7,895 arrayed tumors. Median follow-up was 36.8 months ranging from 1 to 228.7 months. In all patients, PSA values were measured quarterly in the first year, followed by biannual measurements in the second and annual measurements after the third year following surgery. Time to recurrence was defined as the time interval between surgery and first occurrence of a postoperative PSA of ≥0.2 ng/ml and rising thereafter. Patients without evidence of tumor recurrence were censored at the time of the last follow-up. The clinical-pathological features of the arrayed prostate cancers are given in Supplementary Table 3. Deletion status data of 6q15 (*MAP3K7)* (expanded from [23]) and 16q23 (WWOX) [39] were available from previous studies.

The prostate cancer heterogeneity TMA includes 3,170 prostate cancer spots from 317 radical prostatectomy specimens with unifocal prostate cancers according to Wise et al. [40]. The cancers had an average diameter of 68.0 mm (maximum 135 mm). From each of the 317 cancers, 10 different tumor containing tissue blocks were selected for TMA manufacturing. From each of the 10 blocks, one 0.6 mm tumor tissue core was taken, and the 10 tissue cores representing one individual tumor were placed side by side in the TMA block. This resulted in 7 different TMA blocks, each containing 10 tissue cores from 17 to 50 individual tumors. The clinical-pathological features of the arrayed prostate cancers are given in Supplementary Table 4.

Analysis of patient and corresponding histopathological data for research purposes, as well as construction of tissue microarrays from archived diagnostic left-over tissues, was approved by local laws (HmbKHG, §12,1) and by the local ethics committee (Ethics commission Hamburg, WF-049/09 and PV3652). All work was carried out in compliance with the Helsinki Declaration. Additional information is provided in the Supplementary Materials and Methods.

### Fluorescence *in-situ* hybridization

For 6q deletion size determination, three different FISH probe sets were prepared that were analyzed in three adjacent TMA slides. The first consisted of a spectrum green labeled 6q15 *(MAP3K7)* deletion probe (made from BACs RP3-470J8 and RP11-501P02 Source Bioscience, UK) and a spectrum orange labeled commercial centromere 6 probe (#6J36-06; Abbott, Wiesbaden, Germany) as a reference. The second probe set included a spectrum green labeled 6q16 deletion probe (made from BACs RP11-624G20 and RP11-392E05 Source Bioscience, UK), a spectrum orange labeled 6q14 deletion probe (made from BACs RP11-72C17 and RP11-475H7) and a spectrum aqua labeled commercial centromere 6 probe (#6J54-06; Abbott, Wiesbaden, Germany) as a reference. The third probe set was made from a spectrum green labeled 6q22 deletion probe (made from BACs RP11-746B17 and RP11-769C19), a spectrum orange labeled 6q12 deletion probe (made from BACs RP11-473K10 and RP11-707M13) and a spectrum aqua labeled commercial centromere 6 probe (#6J54-06; Abbott, Wiesbaden, Germany) as a reference. The chromosomal localization of these probe sets is shown in Supplementary Figure 4A, 4B.

For 16q deletion size determination, two different FISH probe sets were prepared that were analyzed in two adjacent TMA slides. The first consisted of a spectrum green labeled 16q23 *(WWOX)* deletion probe (made from BACs RP11-190D6 and RP11-345K17) and a spectrum orange labeled commercial centromere 6 probe (#6J36-06; Abbott, Wiesbaden, Germany) as a reference. The second included a spectrum green labeled 16q24 deletion probe (made from BACs RP11-788A09 and RP11-737-K02), a spectrum orange labeled 16q21 deletion probe (made from BACs RP11-575-H07 and RP11-631-D06) and a spectrum aqua labeled commercial centromere 6 probe (#6J54-06; Abbott, Wiesbaden, Germany) as a reference. The chromosomal localization of these probe sets is shown in Supplementary Figure 4D, 4E.

For scoring of FISH, the predominant signal counts of each individual FISH probe were recorded per tissue spot. Heterozygous deletion was defined as presence of fewer locus specific signals than centromere 6 probe signals in >60% of tumor nuclei. Homozygous deletion was assumed if only the centromere signals but no locus specific FISH signals were present in the tumor cells, and if locus specific FISH signals were also visible in adjacent normal cells (tissue spots lacking normal cells were excluded from diagnosis of homozygous deletions). Tumors with complete lack of fluorescence signals in all nuclei (tumor and normal) were regarded as non-interpretable. In addition, tissue samples were excluded if a carefully morphological and immunohistochemical analysis (basal cell marker 34ßE12; AMACR) suggested absence of clear-cut tumor cells in adjacent tissue microarray sections. Representative FISH images of cancers with and without 6q or 16q deletion are shown in Supplementary Figure 4C, 4F. Additional information is provided in the material and method supplementary.

### Determination of the deletion size

Tumors were grouped according to the number of adjacent deleted loci at 6q and 16q according to the following criteria: At 6q, small deletions were assumed if the deletion was limited to 6q15 or 6q14-q16, medium sized deletions were assumed if the deletion was limited to 6q14-q22 (including 6q14-q16), and large deletions were assumed if the deletion was present in all analyzed loci (6q14, 6q15, 6q16, 6q21, 6q22). At 16q, small deletions were assumed if the deletion was limited to 16q24, medium sized deletions were assumed if the deletion was limited to 16q23-q24, and large deletions were assumed if the deletion was present in all analyzed loci (16q21, 16q23, 16q24).

### Cell culture, constructs and lentivirus production

For depletion experiments, five shRNA constructs per gene were tested and the shRNA construct with the highest depletion efficiency was used in this study. shRNA vectors were obtained from Sigma Aldrich (St. Louis, USA), and included lentiviral pLKO.1, as well as constructs from the RNAi Consortium (TRC) vector collection. Overall, 37 potential tumor suppressor genes located between 6q12-22 were depleted in DU145, PC-3 and BPH-1 prostate cell lines (complete list in Supplementary Table 5). Prostate cancer cells were transduced with lentiviruses containing shRNAs directed against Neg and *GFP* (both as a negative control), *PTEN* (not PC-3), *mTOR* (only PC-3) and *RB1* (each as a positive control) and all examined genes. For co-depletion, DU145 cells were transduced with lentiviruses containing 2-4 shRNAs directed against the potential tumor suppressor genes *UBE2J1, ZNF292, SMAP1* and/or *HMGN3* and the negative controls Neg and *GFP* (Supplementary Figure 3A). Transduced target cells were selected with puromycin (1.5 μg/ml). Additional information is provided in the material and method supplementary.

### Western blot analysis and Taqman PCR

Verification of shRNA mediated gene knockdown was perfomed by western blot and Taqman PCR analyses (see material and method supplementary, Supplementary Figure 5 and Supplementary Table 6).

### Colony formation assay

For single knockdown experiments BPH-1, DU145 and PC-3 prostate cells were plated at about 2x10^5^ in 6 well plates. For tumor suppressor gene screening, all randomized selected genes and controls (negative and positive as described above) were analyzed in duplicate. Interesting genes were validated in an additional quadruplicate analysis. Cells were transfected with 4 μg of indicated shRNA construct using Lipofectamine 2000 (Life Technologies, NY, USA) for 36 hours, and cultured in medium containing puromycin (1.5 μg/ml) for at least 2 weeks. The selection medium was renewed every 2-3 days. When drug-resistant colonies became visible they were fixed with methanol and stained with Giemsa and the number of colonies with a diameter ≥1 mm was determined in knockdown versus control cell lines. For multiple knockdown experiments, stable co-transduced DU145 cells were plated at about 1x10^3^ in 6 well plates. Because these stable transduced cells had a high vitality and tended to grow rapidly we decided to measure colony size after one week instead of colony number to quantify tumor cell aggressiveness.

### Soft agar assay

A layer of 0.6% low-melting agarose in standard culture medium was prepared in 6 well plates. On top, a layer of 0.3% agarose containing 1x10^4^ transduced DU145 cells was plated. Transduced cells were depleted for *UBE2J1*, *ZNF292*, *SMAP1*, and *HMGN3*, or combinations of these genes, as well as shNeg and shGFP as negative controls. At day 14, colony size was measured and the average diameter of the colonies was determined.

### Invasion assay

1x10^5^ transduced DU145 cells were re-suspended in 0.5ml of pure RPMI-1640 medium and dispersed in Matrigel Invasion Chambers and Control Inserts (BD BioCoat™, Bedford, USA) placed in 24 well plates. The lower wells contained 0.5ml RPMI-1640 medium supplemented with 10% FCS. Transduced cells were single or co-depleted for *UBE2J1*, *ZNF292*, *SMAP1*, and *HMGN3*, or contained shNeg and shGFP as a negative control. After 24 hours, cells on the surface of the membrane were removed with a cotton swab, fixed with methanol and stained with Giemsa. Cells were counted and the invasion rate (=cell number Invasion chamber/ cell number Contol Insert) and invasion index (=invasion rate probe/invasion rate negative control) was determined.

### Gene set enrichment analysis

The online gene set enrichment analysis (GSEA) software of the Broad Institute (http://www.broadinstitute.org/gsea/msigdb/index.jsp) was queried to search for gene sets that are enriched for expression changes of the candidate tumor suppressor genes identified at 6q and the 16q candidate tumor suppressor genes obtained from the literature.

### Statistic

For statistical analysis, the JMP 9.0 software (SAS Institute Inc., NC, USA) was used. Contingency tables were calculated to study association between 6q and 16q deletion size and clinico-pathological variable, and the Chi-square (Likelihood) test was used to find significant relationships. Kaplan Meier curves were generated for PSA recurrence free survival. The log-Rank test was applied to test the significance of differences between stratified survival functions. Cox proportional hazards regression analysis was performed to test the statistical independence and significance between pathological, molecular, and clinical variables. A p-value of ≤ 0.05 was determined as statistical significant.

## SUPPLEMENTARY MATERIALS FIGURES AND TABLES



## Abbreviations

AR: androgen receptor
CYCLOPS: copy number alterations yielding cancer liabilities owing to partial loss
FISH: fluorescence in-situ hybridization
Mb: mega base
PSA: prostate specific antigen
TMA: tissue microarray
TSG: tumor suppressor gene

## References

[1] Knudson AG (1971). Mutation and cancer: statistical study of retinoblastoma. Proc Natl Acad Sci U S A.

[2] Knudson AG (1991). Overview: genes that predispose to cancer. Mutat Res.

[3] Solimini NL, Xu Q, Mermel CH, Liang AC, Schlabach MR, Luo J, Burrows AE, Anselmo AN, Bredemeyer AL, Li MZ, Beroukhim R, Meyerson M, Elledge SJ (2012). Recurrent hemizygous deletions in cancers may optimize proliferative potential. Science.

[4] Davoli T, Xu AW, Mengwasser KE, Sack LM, Yoon JC, Park PJ, Elledge SJ (2013). Cumulative haploinsufficiency and triplosensitivity drive aneuploidy patterns and shape the cancer genome. Cell.

[5] Berger AH, Pandolfi PP (2011). Haplo-insufficiency: a driving force in cancer. J Pathol.

[6] Taylor BS, Schultz N, Hieronymus H, Gopalan A, Xiao Y, Carver BS, Arora VK, Kaushik P, Cerami E, Reva B, Antipin Y, Mitsiades N, Landers T (2010). Integrative genomic profiling of human prostate cancer. Cancer Cell.

[7] Sun J, Liu W, Adams TS, Li X, Turner AR, Chang B, Kim JW, Zheng SL, Isaacs WB, Xu J (2007). DNA copy number alterations in prostate cancers: a combined analysis of published CGH studies. Prostate.

[8] Williams JL, Greer PA, Squire JA (2014). Recurrent copy number alterations in prostate cancer: an in silico meta-analysis of publicly available genomic data. Cancer Genet.

[9] Berger MF, Lawrence MS, Demichelis F, Drier Y, Cibulskis K, Sivachenko AY, Sboner A, Esgueva R, Pflueger D, Sougnez C, Onofrio R, Carter SL, Park K (2010). The genomic complexity of primary human prostate cancer. Nature.

[10] Weischenfeldt J, Simon R, Feuerbach L, Schlangen K, Weichenhan D, Minner S, Wuttig D, Warnatz HJ, Stehr H, Rausch T, Jäger N, Gu L, Bogatyrova O (2013). Integrative genomic analyses reveal androgen-driven somatic alteration landscape in early-onset prostate cancer. Cancer Cell.

[11] Cooper CS, Eeles R, Wedge DC, Van Loo P, Gundem G, Alexandrov LB, Kremeyer B, Butler A, Lynch AG, Camacho N, Massie CE, Kay J, Luxton HJ (2015). Analysis of the genetic phylogeny of multifocal prostate cancer identifies multiple independent clonal expansions in neoplastic and morphologically normal prostate tissue. Nat Genet.

[12] Hieronymus H, Schultz N, Gopalan A, Carver BS, Chang MT, Xiao Y, Heguy A, Huberman K, Bernstein M, Assel M, Murali R, Vickers A, Scardino PT (2014). Copy number alteration burden predicts prostate cancer relapse. Proc Natl Acad Sci U S A.

[13] Burr ML, Cano F, Svobodova S, Boyle LH, Boname JM, Lehner PJ (2011). HRD1 and UBE2J1 target misfolded MHC class I heavy chains for endoplasmic reticulum-associated degradation. Proc Natl Acad Sci U S A.

[14] Sangar F, Schreurs AS, Umana-Diaz C, Claperon A, Desbois-Mouthon C, Calmel C, Mauger O, Zaanan A, Miquel C, Flejou JF, Praz F (2014). Involvement of small ArfGAP1 (SMAP1), a novel Arf6-specific GTPase-activating protein, in microsatellite instability oncogenesis. Oncogene.

[15] Barkess G, Postnikov Y, Campos CD, Mishra S, Mohan G, Verma S, Bustin M, West KL (2012). The chromatin-binding protein HMGN3 stimulates histone acetylation and transcription across the Glyt1 gene. Biochem J.

[16] Dhar SK, Delmolino L, Dutta A (2001). Architecture of the human origin recognition complex. J Biol Chem.

[17] Frei E, Holden SA, Gonin R, Waxman DJ, Teicher BA (1993). Antitumor alkylating agents: *in vitro* cross-resistance and collateral sensitivity studies. Cancer Chemother Pharmacol.

[18] Nijhawan D, Zack TI, Ren Y, Strickland MR, Lamothe R, Schumacher SE, Tsherniak A, Besche HC, Rosenbluh J, Shehata S, Cowley GS, Weir BA, Goldberg AL (2012). Cancer vulnerabilities unveiled by genomic loss. Cell.

[19] Yip PY, Kench JG, Rasiah KK, Benito RP, Lee CS, Stricker PD, Henshall SM, Sutherland RL, Horvath LG (2011). Low AZGP1 expression predicts for recurrence in margin-positive, localized prostate cancer. Prostate.

[20] Mao X, Boyd LK, Yanez-Munoz RJ, Chaplin T, Xue L, Lin D, Shan L, Berney DM, Young BD, Lu YJ (2011). Chromosome rearrangement associated inactivation of tumour suppressor genes in prostate cancer. Am J Cancer Res.

[21] Huang S, Gulzar ZG, Salari K, Lapointe J, Brooks JD, Pollack JR (2012). Recurrent deletion of CHD1 in prostate cancer with relevance to cell invasiveness. Oncogene.

[22] Krohn A, Seidel A, Burkhardt L, Bachmann F, Mader M, Grupp K, Eichenauer T, Becker A, Adam M, Graefen M, Huland H, Kurtz S, Steurer S (2013). Recurrent deletion of 3p13 targets multiple tumour suppressor genes and defines a distinct subgroup of aggressive ERG fusion-positive prostate cancers. J Pathol.

[23] Kluth M, Hesse J, Heinl A, Krohn A, Steurer S, Sirma H, Simon R, Mayer PS, Schumacher U, Grupp K, Izbicki JR, Pantel K, Dikomey E (2013). Genomic deletion of MAP3K7 at 6q12-22 is associated with early PSA recurrence in prostate cancer and absence of TMPRSS2:ERG fusions. Mod Pathol.

[24] Ishkanian AS, Mallof CA, Ho J, Meng A, Albert M, Syed A, van der Kwast T, Milosevic M, Yoshimoto M, Squire JA, Lam WL, Bristow RG (2009). High-resolution array CGH identifies novel regions of genomic alteration in intermediate-risk prostate cancer. Prostate.

[25] Lapointe J, Li C, Giacomini CP, Salari K, Huang S, Wang P, Ferrari M, Hernandez-Boussard T, Brooks JD, Pollack JR (2007). Genomic profiling reveals alternative genetic pathways of prostate tumorigenesis. Cancer Res.

[26] Liu W, Chang BL, Cramer S, Koty PP, Li T, Sun J, Turner AR, Von Kap-Herr C, Bobby P, Rao J, Zheng SL, Isaacs WB, Xu J (2007). Deletion of a small consensus region at 6q15, including the MAP3K7 gene, is significantly associated with high-grade prostate cancers. Clin Cancer Res.

[27] Dal Bo M, Rossi FM, Rossi D, Deambrogi C, Bertoni F, Del Giudice I, Palumbo G, Nanni M, Rinaldi A, Kwee I, Tissino E, Corradini G, Gozzetti A (2011). 13q14 deletion size and number of deleted cells both influence prognosis in chronic lymphocytic leukemia. Genes Chromosomes Cancer.

[28] Watson JE, Doggett NA, Albertson DG, Andaya A, Chinnaiyan A, van Dekken H, Ginzinger D, Haqq C, James K, Kamkar S, Kowbel D, Pinkel D, Schmitt L (2004). Integration of high-resolution array comparative genomic hybridization analysis of chromosome 16q with expression array data refines common regions of loss at 16q23-qter and identifies underlying candidate tumor suppressor genes in prostate cancer. Oncogene.

[29] Sun X, Frierson HF, Chen C, Li C, Ran Q, Otto KB, Cantarel BL, Vessella RL, Gao AC, Petros J, Miura Y, Simons JW, Dong JT (2005). Frequent somatic mutations of the transcription factor ATBF1 in human prostate cancer. Nat Genet.

[30] Rowley DR, Dang TD, Larsen M, Gerdes MJ, McBride L, Lu B (1995). Purification of a novel protein (ps20) from urogenital sinus mesenchymal cells with growth inhibitory properties *in vitro*. J Biol Chem.

[31] Wang X, Chao L, Ma G, Chen L, Zang Y, Sun J (2011). The prognostic significance of WWOX expression in patients with breast cancer and its association with the basal-like phenotype. J Cancer Res Clin Oncol.

[32] Toyooka KO, Toyooka S, Virmani AK, Sathyanarayana UG, Euhus DM, Gilcrease M, Minna JD, Gazdar AF (2001). Loss of expression and aberrant methylation of the CDH13 (H-cadherin) gene in breast and lung carcinomas. Cancer Res.

[33] Gibbs GM, Roelants K, O’Bryan MK (2008). The CAP superfamily: cysteine-rich secretory proteins, antigen 5, and pathogenesis-related 1 proteins--roles in reproduction, cancer, and immune defense. Endocr Rev.

[34] Schaeffer EM, Marchionni L, Huang Z, Simons B, Blackman A, Yu W, Parmigiani G, Berman DM (2008). Androgen-induced programs for prostate epithelial growth and invasion arise in embryogenesis and are reactivated in cancer. Oncogene.

[35] Mani RS, Tomlins SA, Callahan K, Ghosh A, Nyati MK, Varambally S, Palanisamy N, Chinnaiyan AM (2009). Induced chromosomal proximity and gene fusions in prostate cancer. Science.

[36] Xue W, Kitzing T, Roessler S, Zuber J, Krasnitz A, Schultz N, Revill K, Weissmueller S, Rappaport AR, Simon J, Zhang J, Luo W, Hicks J (2012). A cluster of cooperating tumor-suppressor gene candidates in chromosomal deletions. Proc Natl Acad Sci U S A.

[37] Birnbaum D, Adelaide J, Popovici C, Charafe-Jauffret E, Mozziconacci MJ, Chaffanet M (2003). Chromosome arm 8p and cancer: a fragile hypothesis. Lancet Oncol.

[38] El Gammal AT, Bruchmann M, Zustin J, Isbarn H, Hellwinkel OJ, Kollermann J, Sauter G, Simon R, Wilczak W, Schwarz J, Bokemeyer C, Brummendorf TH, Izbicki JR (2010). Chromosome 8p deletions and 8q gains are associated with tumor progression and poor prognosis in prostate cancer. Clin Cancer Res.

[39] Kluth M, Runte F, Barow P, Omari J, Abdelaziz ZM, Paustian L, Steurer S, Christina Tsourlakis M, Fisch M, Graefen M, Tennstedt P, Huland H, Michl U (2015). Concurrent deletion of 16q23 and PTEN is an independent prognostic feature in prostate cancer. Int J Cancer.

[40] Wise AM, Stamey TA, McNeal JE, Clayton JL (2002). Morphologic and clinical significance of multifocal prostate cancers in radical prostatectomy specimens. Urology.

