# Supplementary material for: Deletion lengthening at chromosomes 6q and 16q targets multiple tumor suppressor genes and is associated with an increasingly poor prognosis in prostate cancer
Source: Oncotarget. 2017 Nov 11;8(65):108923–35. doi: 10.18632/oncotarget.22408 (PMC5752492; doi:10.18632/oncotarget.22408)
Supplement: Supplementary file 1 [file oncotarget-08-108923-s001.pdf]

Deletion lengthening at chromosomes 6q and 16q targets multiple tumor suppressor genes and is associated with an increasingly poor prognosis in prostate cancer

SUPPLEMENTARY MATERIALS

MATERIALS AND METHODS

Patients and tissue microarrays

All cancer samples were obtained from patients undergoing surgery at the Department of Urology, and the Martini Clinic, Prostate Cancer Center, University Medical Center Hamburg-Eppendorf between 1992 and 2012. The TMA manufacturing process was described earlier in detail [1]. In brief, one 0.6 mm core was taken from a representative tissue block from each patient. The tissues were distributed among different TMA blocks containing up to 522 tumor samples each. Presence or absence of cancer tissue was validated by immunohistochemical AMACR and 34BE12 analysis on adjacent TMA sections for all TMAs.

Fluorescence *in situ* hybridization

Four micrometer TMA sections were used for fluorescence *in situ* hybridization (FISH). TMA sections were de-waxed, air-dried, and dehydrated in 70%, 85%, and 100% ethanol. Slides were pretreated in VP 2000 Pretreatment Reagent (Abbott, Des Plaines, USA) for 15 min at 80°C, followed by 150 min incubation at 37°C in 0.5% protease 1 solution (Abbott, Des Plaines, USA). 4 µl of FISH probe mix (as described below) in 70% formamide 2x SSC solution was applied to the slides and co-denatured with the cellular DNA in a Hybrite hybridization oven for 10 min at 72°C and hybridization over night at 37°C. After hybridization, slides were subjected to serial stringent washings (2x SSC solution with 0,3% NP40 at 72 °C for 2 minutes) and counterstained with 0.2µmol/L 4'-6-diamidino-2-phenylindole in antifade solution.

Western blot analysis

Protein were isolated from transduced prostate cell lines with cell extraction buffer (Invitrogen, Carlsbach, United States) and measured with Qubit 2.0 Fluoremeter (Invitrogen, Carlsbach, United States). For Western Blot analysis 40µg or 60µg (only UBE2J1 in DU145) Protein was loaded on Mini-PROTEAN® TGX™ Gels (Bio-Rad, Hercules, United States), and then transferred to 0.2µm nitrocellulose membranes (Bio-Rad, Hercules, United States) by western blotting. For Protein detection following antibodies were used: anti-alpha-tubulin (Sigma-Aldrich, Saint Louis, United States), anti-UBE2J1 (Sigma-Aldrich, Saint Louis, United States), anti-ZNF292 (Abcam, Cambridge, United Kingdom), anti-HMGN3 (Novus Biologicals, Cambridge, United Kingdom), anti-SMAP1 (Novus Biologicals, Cambridge, United Kingdom), anti-ORC3 (LSBio, Seattle, United States) and anti-PM20D2 (Abgent, San Diego, United States) (Supplementary Figure 5a).

RNA-isolation and Taqman PCR

Total RNA was extracted using Trizol and the NucleoSpin RNA2 (Macherey-Nagel, Düren, Germany). RNA was reverse transcribed using the High Capacity cDNA Archive Kit (Applied Biosystems, Darmstadt, Germany). Real time reverse transcriptase-polymerase chain reaction (RT-PCR) was performed as described previously [2]. For all genes, Assay-on-Demand primer/probe sets were obtained from Applied Biosystems (Assay IDs are available upon request). Relative expression was calculated by normalization to a housekeeper mRNA (GAPDH) by the standard curve method and were standardized to 10^4 GAPDH copy numbers [3] (Supplementary Figure 5b and Supplementary Table 6).

Cell culture, constructs and lentivirus production

The BPH-1, PC-3 and DU145 prostate cell lines were obtained and cultured according to the supplier's instructions (DSMZ, Braunschweig, Germany, ATCC, Manassas, USA). All cell lines have been periodically tested (every 3 months) for cell morphology, growth rate and gene expression. The shRNAs used for depletion of 6 relevant genes identified in this study (*UBE2J1, ZNF292, SMAP1, HMGN3, ORC3, PM20D2*) and controls (*PTEN, mTOR, RB1, GFP, shNeg*) included the following mature sense sequences: *UBE2J1*: ACATTCTGCATTGGGTATAAT, *ZNF292*: TGAAGGCTGTGACCGTATATA, *SMAP1*: CCGATGATTTCTAATCCCTTA, *HMGN3*: TCTGCC AGATTGTCAGCGAAA, *ORC3*: GCTACTTCTTAC AACTCAGTT, *PM20D2*: CCAGATATGGCTGAACA TGAT, *PTEN*: CCACAGCTAGAACTTATCAAA [4], *mTOR*: TCAGCGTCCCTACCTTCTTCT [5], *RB1*:

GTGCGCTCTTGAGGTTGTAAT [4], *green fluorescent protein* (*GFP*) CAACAAGATGAAGAGCACCAA [6] and E. coli polymerase I (shNeg): TTATC GCGCATATCACGCG [7]. Lentivirus supernatants were prepared after co-transfection of a lentivirus vector plasmid with pVSV-G (expressing the VSV envelope protein), pREV and pRRE (expressing lentivirus helper functions) into HEK-293T cells as described previously [8].

REFERENCES

1. Kononen J, Bubendorf L, Kallioniemi A, Barlund M, Schraml P, Leighton S, Torhorst J, Mihatsch MJ, Sauter G, Kallioniemi OP. Tissue microarrays for high-throughput molecular profiling of tumor specimens. Nature Med. 1998; 4:844-847.
2. Livak KJ, Schmittgen TD. Analysis of relative gene expression data using real-time quantitative PCR and the 2(-Delta Delta C(T)) method. Methods. 2001; 25:402-408.
3. Bookout AL, Mangelsdorf DJ. Quantitative real-time PCR protocol for analysis of nuclear receptor signaling pathways. Nucl Recept Signal. 2003; 1:e012.
4. Kluth M, Hesse J, Heinl A, Krohn A, Steurer S, Sirma H, Simon R, Mayer PS, Schumacher U, Grupp K, Izbicki JR, Pantel K, Dikomey E, et al. Genomic deletion of MAP3K7 at 6q12-22 is associated with early PSA recurrence in prostate cancer and absence of TMPRSS2:ERG fusions. Mod Pathol. 2013; 26:975-983.
5. Sarbassov DD, Guertin DA, Ali SM, Sabatini DM. Phosphorylation and regulation of Akt/PKB by the rictor-mTOR complex. Science. 2005; 307:1098-1101.
6. Everett RD, Rechter S, Papior P, Tavalai N, Stamminger T, Orr A. PML contributes to a cellular mechanism of repression of herpes simplex virus type 1 infection that is inactivated by ICP0. J Virol. 2006; 80:7995-8005.
7. Glass M, Everett RD. Components of promyelocytic leukemia nuclear bodies (ND10) act cooperatively to repress herpesvirus infection. J Virol. 2013; 87:2174-2185.
8. Cuchet D, Sykes A, Nicolas A, Orr A, Murray J, Sirma H, Heeren J, Bartelt A, Everett RD. PML isoforms I and II participate in PML-dependent restriction of HSV-1 replication. J Cell Sci. 2011; 124:280-291.
9. Taylor BS, Schultz N, Hieronymus H, Gopalan A, Xiao Y, Carver BS, Arora VK, Kaushik P, Cerami E, Reva B, Antipin Y, Mitsiades N, Landers T, et al. Integrative genomic profiling of human prostate cancer. Cancer Cell. 2010; 18:11-22.
10. Huang S, Gulzar ZG, Salari K, Lapointe J, Brooks JD, Pollack JR. Recurrent deletion of CHD1 in prostate cancer with relevance to cell invasiveness. Oncogene. 2012; 31:4164-4170.
11. Mao X, Boyd LK, Yanez-Munoz RJ, Chaplin T, Xue L, Lin D, Shan L, Berney DM, Young BD, Lu YJ. Chromosome rearrangement associated inactivation of tumour suppressor genes in prostate cancer. Am J Cancer Res. 2011; 1:604-617.
12. Krohn A, Seidel A, Burkhardt L, Bachmann F, Mader M, Grupp K, Eichenauer T, Becker A, Adam M, Graefen M, Huland H, Kurtz S, Steurer S, et al. Recurrent deletion of 3p13 targets multiple tumour suppressor genes and defines a distinct subgroup of aggressive ERG fusion-positive prostate cancers. J Pathol. 2013; 231:130-141.
13. Mader M, Simon R, Steinbiss S, Kurtz S. FISH Oracle: a web server for flexible visualization of DNA copy number data in a genomic context. J Clin Bioinforma. 2011; 1:20.
14. Mader M, Simon R, Kurtz S. FISH Oracle 2: a web server for integrative visualization of genomic data in cancer research. J Clin Bioinforma. 2014; 4:5.

Supplementary Table 1: Effect of co-depletion of candidate tumor suppressor genes in DU145 cells in the invasion assay

Depletion probe	Invasion rate (%)	Invasion index
Neg	50.00	1.04
shRB1	54.81	1.15
shZNF292	46.75	1.03
shUBE2J1	49.69	1.11
shHMGN3	66.77	1.48
shSMAP1	95.60	1.92
shUBE2J1, shZNF292	46.29	1.01
shUBE2J1, shHMGN3	43.19	0.80
shUBE2J1, shSMAP1	51.48	1.23
shSMAP1, shHMGN3	157.54	2.89
shUBE2J1, shZNF292, shSMAP1	163.76	1.91
shUBE2J1, shSMAP1, shHMGN3	182.42	3.53
shUBE2J1, shHMGN3, shZNF292	83.87	1.79
shUBE2J1, shHMGN3, shSMAP1, shZNF292	141.23	3.16

Supplementary Table 2: Multivariate analysis (Cox regression) including clinical and pathological parameters in addition to the deletion size

	Parameter	RR	95% CI	P-value
Tumor stage	pT2 vs pT3a	1.69	1.10-2.62	0.0011
	pT3a vs pT3b	1.24	0.82-1.87	
	pT3b vs pT4	2.80	1.07-6.44	
Gleason grade	≤3+3 vs 3+4	3.16	1.85-5.70	<0.0001
	3+4 vs 4+3	1.96	1.30-2.90	
	4+3 vs ≥4+4	0.73	0.27-1.64	
Nodal stage	pN0 vs pN+	2.72	1.62-4.42	0.0003
PSA level	<4 vs 4-10	0.77	0.41-1.55	0.1547
	4-10 vs 10-20	1.06	0.71-1.59	
	10-20 vs >20	1.55	1.00-2.40	
R status	R0 vs R1	1.37	0.07-1.91	0.0710
deletion size	no deletion vs 1-20 Mb	1.02	0.69-1.49	0.0003
	1-20 Mb vs 30-40 Mb	2.64	1.54-4.39	
	30-40 Mb vs >40 Mb	0.99	0.45-2.04	

Supplementary Table 3: Pathological and clinical data of the arrayed prostate cancers at the prostate cancer prognosis TMA. Percent in the column "Study cohort on TMA" refers to the fraction of samples across each category. Percent in column "Biochemical relapse among categories" refers to the fraction of samples with biochemical relapse within each parameter in the different categories.

	No. of patients (%)	
	Study cohort on TMA (n=7482)	Biochemical relapse among categories (n=1457)
**Follow-up (mo)**		
Mean	53.4	-
Median	36.8	-
**Age (y)**		
≥50	234 (3.2%)	43 (18.4%)
50-60	1912 (25.8%)	368 (19.2%)
60-70	4438 (59.9%)	872 (19.6%)
>70	822 (11.1%)	172 (20.9%)
**Pretreatment PSA (ng(ml)**		
<4	976 (13.2%)	125 (12.8%)
4-10	4443 (60.3%)	650 (14.6%)
10-20	1461 (19.8%)	411 (28.1%)
>20	488 (6.6%)	248 (50.8%)
**pT category (AJCC 2002)**		
pT2	4927 (66.2%)	460 (9.3%)
pT3a	1650 (22.2%)	477 (28.9%)
pT3b	803 (10.8%)	472 (58.8%)
pT4	58 (0.8%)	48 (82.8%)
**Gleason grade**		
≤3+3	2316 (31.2%)	171 (7.4%)
3+4	3804 (51.2%)	693 (18.2%)
4+3	1018 (13.7%)	448 (44.0%)
≥4+4	287 (3.9%)	144 (50.2%)
**pN category**		
pN0	3963 (92.4%)	919 (23.2%)
pN+	328 (7.6%)	207 (63.1%)
**Surgical margin**		
Negative	5921 (80.6%)	914 (15.4%)
Positive	1428 (19.4%)	516 (36.1%)

NOTE: Numbers do not always add up to 7 482 in the different categories because of cases with missing data.
AJCC, American Joint Committee on Cancer.

Supplementary Table 4: Pathological and clinical data of the arrayed prostate cancers of the prostate cancer heterogeneity TMA. Percent in the column "Study cohort on TMA" refers to the fraction of samples across each category

	**Study cohort on TMA (n=317)**
**Age (y)**	
≤50	2 (0.6%)
51-59	89 (28.2%)
60-70	168 (53.2%)
>70	57 (18.0%)
**pT category (AJCC 2002)**	
pT2	89 (28.1%)
pT3a	97 (30.6%)
pT3b	129 (40.7%)
pT4	2 (0.6%)
**Gleason grade**	
≤3+3	4 (1.3%)
3+4	154 (48.6%)
4+3	102 (32.2%)
≥4+4	57 (18.0%)
**pN category**	
pN0	158 (74.2%)
pN+	55 (25.8%)
**Surgical margin**	
negative	175 (57.0%)
positive	132 (43.0%)

NOTE: Numbers do not always add up to 317 in the different categories because of cases with missing data.
AJCC, American Joint Commitee on Cancer.

Supplementary Table 5: shRNA constructs for shRNA-mediated gene knockdown for screening of potential tumor suppressor genes (Sigma Aldrich, St. Louis, USA)

Gensymbol	TRC-Nummer	siRNA-Sequenz
AKRIN2	TRCN0000219960	TTGCAAGAGGTCCCAATTATG
ANKRD6	TRCN0000160562	CTAATCAACAAGCTGGAGAAT
ASF1A	TRCN0000074270	GTGAAGAATACGATCAAGTTT
ATG5	TRCN0000151963	CCTGAACAGAATCATCCTTAA
BACH2	TRCN0000018166	CCTGTAGATCAAATCACAGAT
C6orf162	TRCN0000159717	GCTCTTCATTAAACCTAACAA
C6orf168	TRCN0000140766	GATTCGGATGTGGACATGGAT
CASP8AP2	TRCN0000061766	GCCAATTTACAAATCTGACAA
CCNC	TRCN0000020189	GCATCCAAAGTAGAGGAATTT
CD164	TRCN0000057555	GCTATTGTTCACATAACTCAA
COQ3	TRCN0000035340	CCTGAAACACTAGAGAGCATT
EEF1A1	TRCN0000029333	CCTCTCCAGGATGTCTACAAA
EPHA7	TRCN0000195051	CGATGTGACCTACAGAATA
FBXL4	TRCN0000118341	GCCAGGACTATGTGGAACTTA
GFP	Everett et al.	CAACAAGATGAAGAGCACCAA
HACE1	TRCN035693417	GCCAGTACCTAAAGATTCTAA
HDAC2	TRCN035694819	CAGTCTCACCAATTTCAGAAA
HMGN3	TRCN0000275883	TCTGCCAGATTGTCAGCGAAA
KIAA0776	TRCN0000122062	CCAGTAAGCATAAGTCATATT
LYRM2	TRCN0000230470	GATGATTACTCAAGGCAATAT
MANEA	TRCN0000049651	GCCTCTGAACTTAACTTGGAT
MAP3K7	TRCN035691556	CAGTGTGTCTTGTGATGGAAT
MARCKS	TRCN0000029041	GAGCGGCTTCTCCTTCAAGAA
MMS22L	TRCN0000130019	GCTTCATGGATTACTCTTGTA
mTOR	Sarbassov et al.	TCAGCGTCCCTACCTTCTTCT
NDUFAF4	TRCN0000122686	GCGAGAGCAGATTAGTCTCTA
Neg	Everett et al.	TTATCGCGCATATCACGCG
ORC3	TRCN0000150842	GCTACTTCTTACAACTCAGTT
PHIP	TRCN0000130643	CCATGATATGCCTGACGTTAT
PM20D2	TRCN0000148358	CCAGATATGGCTGAACATGAT
PNISR	TRCN0000156518	GCTCAGGTAGTAGTCGTACTT
PNRC1	TRCN0000063278	GCAAATATAACTTGCCACTAA
PTEN	TRCN035692746	CCACAGCTAGAACTTATCAAA
RARS2	TRCN0000045346	GTACTGGTCAAAGGACTGTAA
RB1	TRCN0000295892	GTGCGCTCTTGAGGTTGTAAT
RRAGD	TRCN0000059537	CCAGGGCCTACAAAGTGAATA
SENP6	TRCN0000272788	CCTTGATCCTCCGGCAAATAT
SLC35A1	TRCN0000038200	CCATCGTTAGTGTATGCTGTT
SMAP1	TRCN0000150993	CCGATGATTTCTAATCCCTTA
TRAF3IP2	TRCN0000160964	GCTTCAGAACACTCATGTCTA
TTK	TRCN035696357	CCAGTTGTAAAGAATGACTTT
UBE2J1	TRCN0000320501	ACATTCTGCATTGGGTATAAT
ZNF292	TRCN0000230618	TGAAGGCTGTGACCGTATATA

Supplementary Table 6: Expression of all estimated 6q genes in BPH-1, DU 145 and PC-3 cell lines. mRNA Expression were measured by TaqMan PCR and calculated with the standard curve method as relative mRNA copy number against 10^4 GAPDH copy numbers

	BPH-1	DU 145	PC-3
	Relative copy number	Relative copy number	Relative copy number
AKIRIN2	170.14	116.53	83.69
ANKRD6	13.62	14.53	1.43
ASF1A	115.36	151.59	195.71
ATG5	66.42	60.38	56.56
BACH2	5.38	0.27	4.22
C6orf162	47.60	36.57	47.43
C6orf168	2.91	49.67	5.09
CASP8AP2	27.50	9.64	6.64
CCNC	168.62	63.97	49.53
CD164	401.17	415.10	544.48
COQ3	28.83	9.89	10.00
EEF1A1	13061.32	10909.35	23414.16
FBXL4	39.92	150.19	42.22
HACE1	9.84	7.99	22.19
HDAC2	157.09	124.63	218.36
HMGN3	114.81	399.85	209.20
KIAA0776	125.54	64.40	47.31
LYRM2	6.51	7.89	4.30
MANEA	72.73	52.54	17.99
MAP3K7	36.48	27.87	17.27
MARCKS	405.71	171.35	19.35
MM2SSL	148.45	28.27	23.70
NDUFAF4	104.53	32.08	65.03
ORC3	125.07	59.56	58.62
PHIP	48.28	62.04	125.16
PM20D2	117.89	100.85	31.33
PNISR	677.98	557.42	311.84
PNRC1	38.53	159.17	50.87
RARS2	54.42	17.28	23.30
RRAGD	5.73	110.69	11.06
SENP6	106.90	64.83	148.08
SLC35A1	378.61	145.79	350.80
SMAP1	113.62	103.12	212.17
TRAF3IP2	104.08	25.99	79.10
TTK	23.77	19.78	36.98
UBE2J1	224.05	275.79	131.26
ZNF292	122.62	95.09	46.40

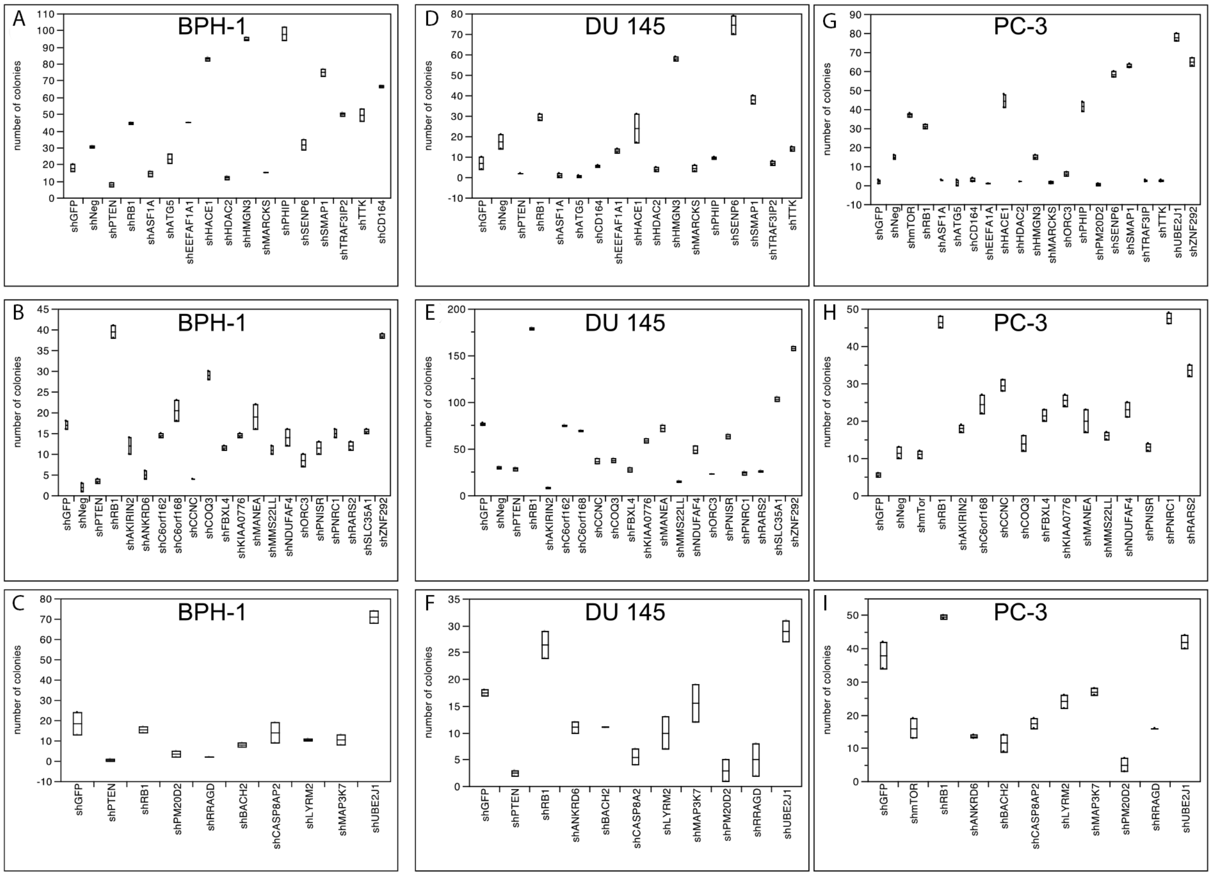

Supplementary Figure 1: Results of all 37 shRNA-mediated gene knockdowns by shRNA transfection at colony formation in **(A-C)** BPH-1, **(D-F)** DU 145, and **(G-I)** PC-3 cells.

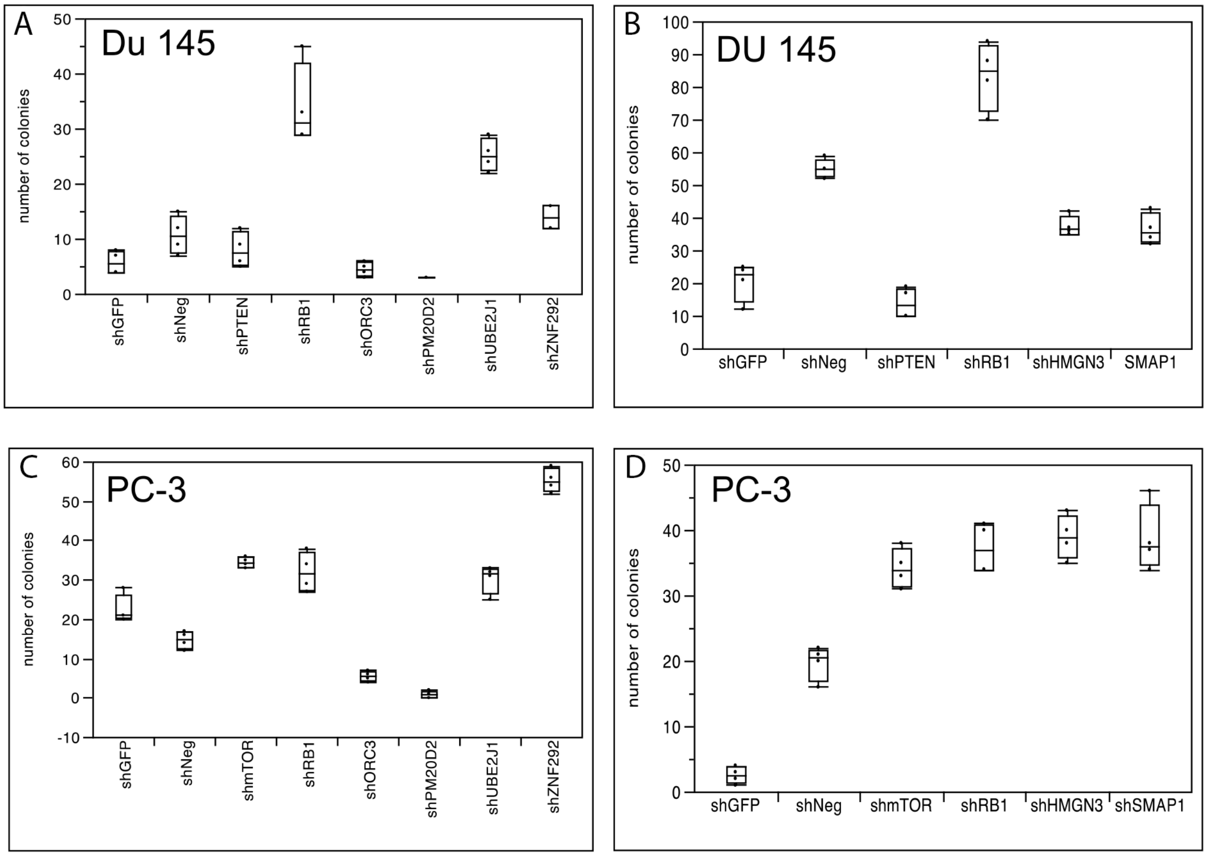

Supplementary Figure 2: Colony formation assay validation of tumor suprressor gene screening by shRNA-mediated depletion of UBE2J1, ZNF292, SMAP1, HMGN3, PM20D2 and ORC3 in **(A-B)** DU 145, and **(C-D)** PC-3 cells. Used controls were shPTEN, shRB1 (both as positive control), shNeg and shGFP (both as negative control).

A

experiment number	shUBE2J1 (µg)	shZNF292 (µg)	shSMAP1 (µg)	shHMGN3 (µg)	shNeg (µg)	shGFP (µg)
1	6.0				3.0	3.0
2		6.0			3.0	3.0
3				6.0	3.0	3.0
4			6.0		3.0	3.0
5	2.5	3.5			3.0	3.0
6	3.0		3.0		3.0	3.0
7	3.0			3.0	3.0	3.0
8	1.5	2.5	2.0		6.0	
9	1.5	2.5		2.0	6.0	
10	2.5	3.5	2.75	3.25		
11			1.5	4.5	3.0	3.0
12	2.0		2.0	2.0	6.0	
13		4.5	1.5		3.0	3.0
14		3.5		2.5	3.0	3.0
15		4.0	1.0	1.0	6.0	
16					6.0	6.0

B

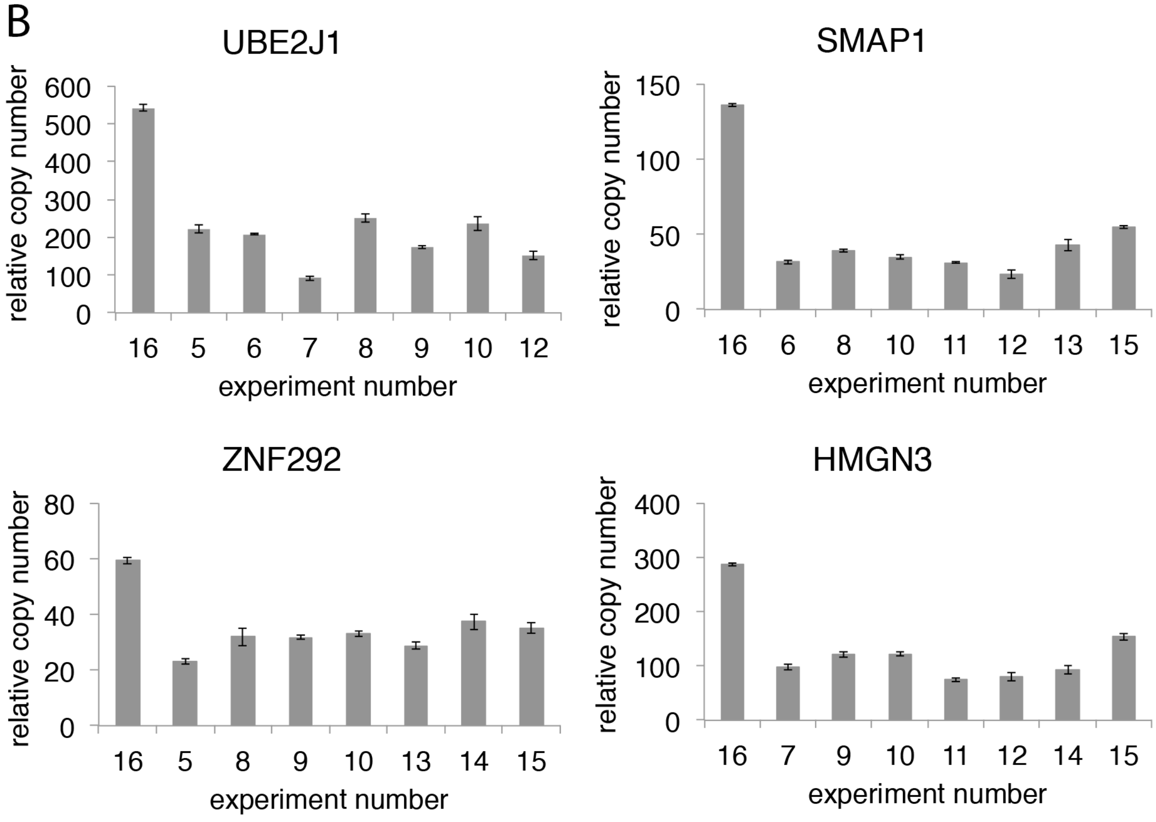

Supplementary Figure 3: Co-depletion of 6q candidate tumor suppressor genes. (A) Composition of shRNA constructs for lentiviral vector production. **(B)** Verification of co-depletion at mRNA level measured by TaqMan PCR and calculated with the standard curve method as relative mRNA copy number against 10^4 GAPDH copy numbers.

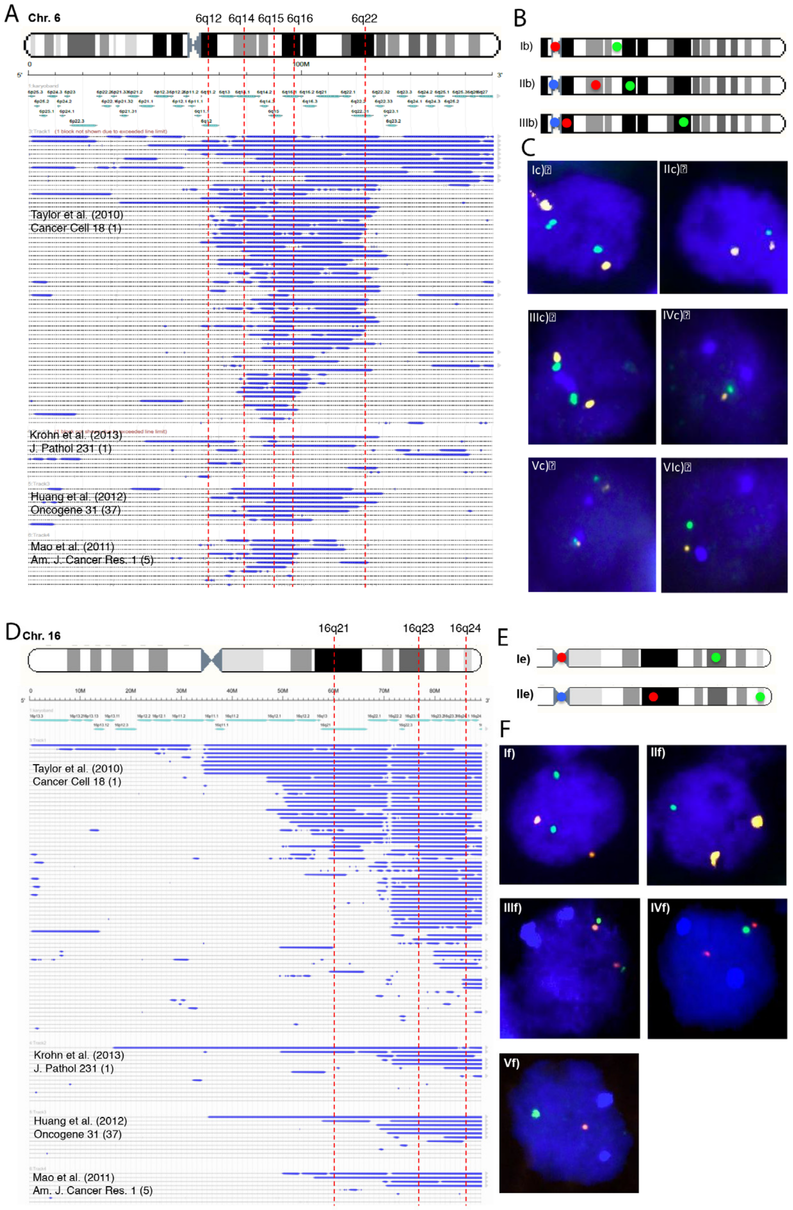

Supplementary Figure 4: Analysis of array-based copy number analysis data. Published prostate cancer copy number data were obtained from previous publications, including 207 datasets from Taylor et al. [9], 86 datasets from Huang et al. [10], 77 datasets from Mao et al. [11], and 72 datasets from our own group [12]. A self-made genomic browser (FISH Oracle) [13, 14] was used re-process the data in order to map all 6q and 16q deletions to the human genome reference sequence (Archive EnsEMBL release 54 – May 2009). A global intensity threshold of -0.3, corresponding to the ratio of the signal intensity ratio between segments with copy number deviations in comparison to the overall intensity across all segments, was selected to define **(A)** 6q and **(D)** 16q deletions in all datasets. Each bar represents the deleted area in a single tumor, red lines mark localization of FISH probes. Compositon and localization of FISH probes for deletion size analysis of **(B)** 6q12-22, and **(E)** 16q21-24. Examples of FISH findings using the different deletion probes for **(C)** 6q12-22, and **(F)** 16q21-24. Ic) Normal 6q15 copy numbers as indicated by two green 6q15 signals and two orange centromere 6 signals. IIc) Heterozygous deletion as indicated by the lack of one green 6q15 signal and two orange centromer 6 signals. IIIc) Normal 6q14 and 6q16 copy numbers as indicated by two green 6q16 signals, two orange 6q14 signals and two aqua centromere 6 signals. IVc) Heterozygous deletion as indicated by the lack of one green 6q16 and one orange 6q14 signal and two aqua centromer 6 signals. Vc) Normal 6q12 and 6q22 copy numbers as indicated by two green 6q22 signals, two orange 6q12 signals and two aqua centromere 6 signals. IVc) Heterozygous deletion as indicated by the lack of one green 6q22 and one orange 6q12 signal and two aqua centromer 6 signals. If) Normal 16q23 copy numbers as indicated by two green 16q23 signals and two orange centromere 6 signals. IIf) Heterozygous deletion as indicated by the lack of one green 16q23 signal and two orange centromer 6 signals. IIIf) Normal 16q21 and 16q24 copy numbers as indicated by two green 16q24 signals, two orange 16q21 signals and two aqua centromere 6 signals. IVf) Heterozygous deletion as indicated by the lack of one green 16q24 and one orange 16q21 signal and two aqua centromer 6 signals.

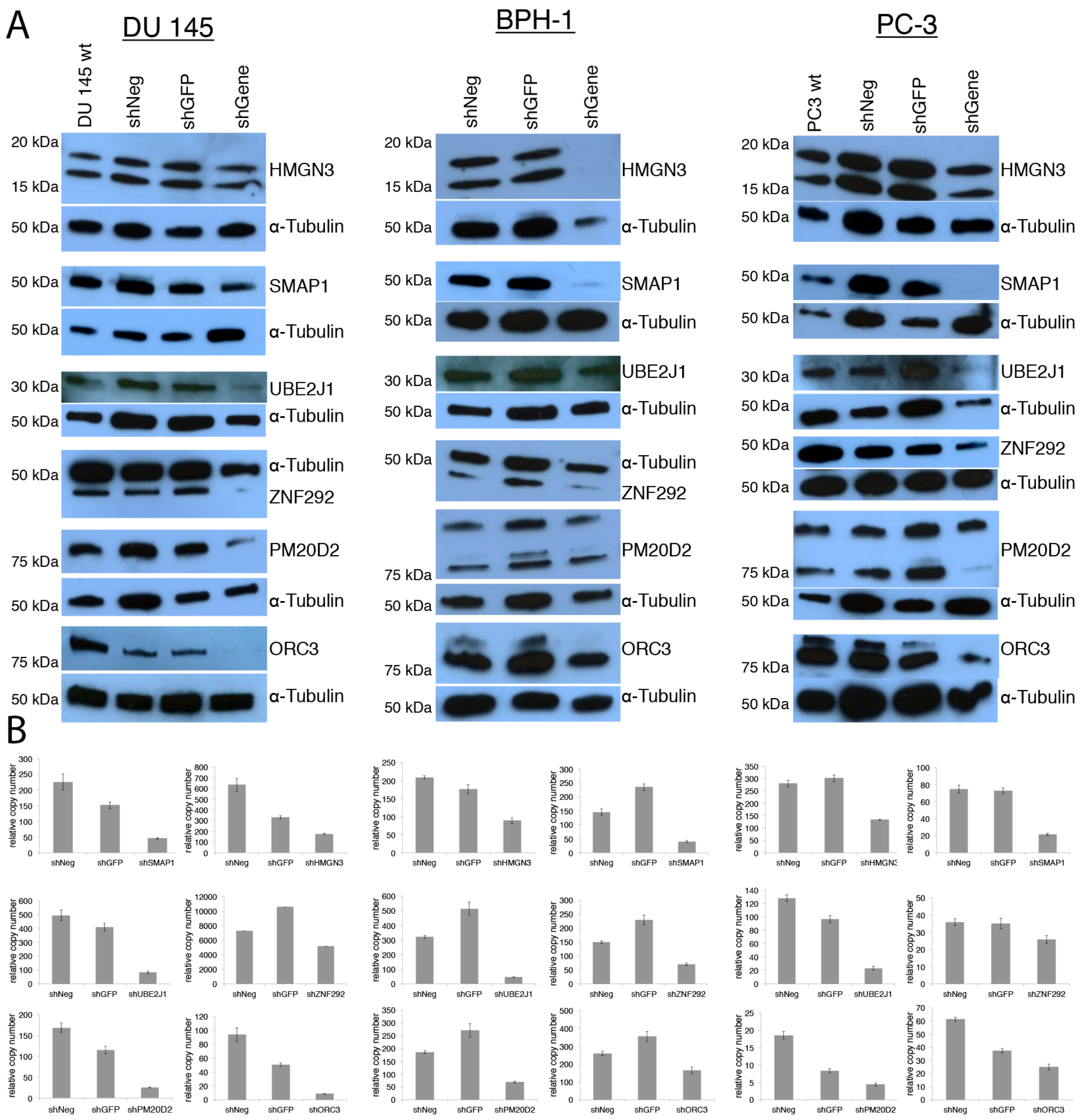

Supplementary Figure 5: Verification of shRNA mediated depletion of the four 6q candidate tumor suppressor genes and the two 6q potential essential genes **(A)** at protein level measured by western blotting and the housekeeper α-Tubulin, **(B)** at mRNA level measured by TaqMan PCR and calculated with the standard curve method as relative mRNA copy number against 10^4 GAPDH copy numbers.
